# Immune System, Microbiota, and Microbial Metabolites: The Unresolved Triad in Colorectal Cancer Microenvironment

**DOI:** 10.3389/fimmu.2021.612826

**Published:** 2021-03-26

**Authors:** Michelle Hanus, Daniela Parada-Venegas, Glauben Landskron, Ana Maria Wielandt, Claudia Hurtado, Karin Alvarez, Marcela A. Hermoso, Francisco López-Köstner, Marjorie De la Fuente

**Affiliations:** ^1^ Laboratory of Innate Immunity, Program of Immunology, Faculty of Medicine, Institute of Biomedical Sciences, Universidad de Chile, Santiago, Chile; ^2^ Research Core, Academic Department, Clínica Las Condes, Santiago, Chile; ^3^ Cancer Center, Clínica Universidad de los Andes, Santiago, Chile

**Keywords:** colorectal cancer, tumor micronvironment (TME), intestinal microbiota, diet-derived metabolites, immune system

## Abstract

Colorectal cancer (CRC) is one of the most common cancers worldwide. As with other cancers, CRC is a multifactorial disease due to the combined effect of genetic and environmental factors. Most cases are sporadic, but a small proportion is hereditary, estimated at around 5-10%. In both, the tumor interacts with heterogeneous cell populations, such as endothelial, stromal, and immune cells, secreting different signals (cytokines, chemokines or growth factors) to generate a favorable tumor microenvironment for cancer cell invasion and metastasis. There is ample evidence that inflammatory processes have a role in carcinogenesis and tumor progression in CCR. Different profiles of cell activation of the tumor microenvironment can promote pro or anti-tumor pathways; hence they are studied as a key target for the control of cancer progression. Additionally, the intestinal mucosa is in close contact with a microorganism community, including bacteria, bacteriophages, viruses, archaea, and fungi composing the gut microbiota. Aberrant composition of this microbiota, together with alteration in the diet‐derived microbial metabolites content (such as butyrate and polyamines) and environmental compounds has been related to CRC. Some bacteria, such as *pks+ Escherichia coli* or *Fusobacterium nucleatum*, are involved in colorectal carcinogenesis through different pathomechanisms including the induction of genetic mutations in epithelial cells and modulation of tumor microenvironment. Epithelial and immune cells from intestinal mucosa have Pattern-recognition receptors and G-protein coupled receptors (receptor of butyrate), suggesting that their activation can be regulated by intestinal microbiota and metabolites. In this review, we discuss how dynamics in the gut microbiota, their metabolites, and tumor microenvironment interplays in sporadic and hereditary CRC, modulating tumor progression.

## Introduction

Colorectal cancer (CRC) is the third most common cancer in both sex worldwide with an estimated 1.8 million new cases and mortality rates in 2018 over 800,000 (1). The vast majority of CRC cases are classified as sporadic and occur in average risk patients, with no family history or an apparent genetic predisposition demonstrated, mostly affecting people older than 50 (2). However, heredity represents a significant cause of CRC, in which 20-30% are familial cases, with 5-10% linked to inherited variants in cancer-predisposing genes (3). The most common hereditary CRC is Lynch syndrome, accounting for 2-3% of cases, followed by familial adenomatous polyposis (FAP) responsible for 0.5-1% of cases, and other hereditary variants such as MUTYH-associated polyposis (MAP), Peutz-Jeghers syndrome, among others, represent <1% of cases (3).

Diverse studies have highlighted the role of gut commensal microbiota in host physiology and diseases such as cancer by modulating the immune response, genetic damage, and apoptosis (4–6). The intestinal tract is an extensive surface in contact with lumen antigens, including more than 100 trillion microorganisms, predominating the phyla Firmicutes and Bacteroidetes (7). Accordingly, the mucosa has a complex immune system maintaining the homeostasis in steady conditions but swiftly responding to an insult (8). Additionally, alteration in the gut microbiota disrupts the epithelial barrier promoting inflammation and tumorigenesis-associated pathways (9).

Cancer, rather than being formed by homogeneous malignant cells, contains a heterogeneous cell population, such as endothelial, stromal, and immune cells, secreting soluble signals (cytokines, chemokines, or growth factors), interacting with tumor cells, generating a favorable microenvironment to support tumor growth and progression (10, 11). Moreover, tumor and microenvironment cells respond to signals from microbiota, regulating multiple host pathways related to carcinogenic processes, such as the induction of mutations in infected cells by a pathogenic *E. coli* strain or epithelial invasion by *Fusobacterium nucleatum* (12, 13). Additionally, microbiota, through the production of metabolites, such as short-chain fatty acids (SCFAs) or polyamines (4, 14), controls tumor cell function and microenvironment.

Here we aim to review the role of tumor microenvironment components in sporadic and hereditary CRC, with an emphasis in interactions between microbiota, immune system, and diet-derived metabolites.

## Immune System in Tumor Microenvironment

The tumor microenvironment (TME) has a wide diversity of molecules and cell types, including immune cells, fibroblast, adipocytes, endothelial cell and microbiome (6, 15). The immune system is particularly composed of innate immune cells such as neutrophils, macrophages, dendritic cells (DCs), mast cells and natural killer cells (NK), and adaptive immune cells such as T and B lymphocytes (16), participating in prevention and promotion of tumor development, having pro and anti-tumor functions (17).

Immunosurveillance refers to the role of the immune system in recognizing antigens from transformed cells, thus generating memory and effector cells, which seek out and control generation of new tumor cells (18).

Tumor cells undergo a selection process called immunoediting consisting of 3 stages: elimination, equilibrium, and escape. In the first phase, the antitumor immune response eliminates initiating tumor cells; the tumor then evolves into a static phase (equilibrium) in which some malignant cells avoid the immune response, without tumor elimination. Finally, the resistant clones manage to evade the immune system acquiring pro-tumorigenic properties and reducing their immunogenicity, allowing tumor development and clinical manifestations (19).

Lymphocytes are key cells in the tumor microenvironment, and according to their profile have different functions in cancer progression: CD8^+^ T cells lyse tumor cells and release cytokines enhancing cytotoxic responses, such as IFNγ; CD4^+^ Th1 T cells release cytokines improving lymphocyte cytotoxic function; regulatory CD4^+^ T cells (Treg) are immunosuppressive cells, preventing chronic inflammation and maintaining immune tolerance, by suppressing effector T cell proliferation and activation (17, 20). B cells recognize tumor antigens and produce specific antibodies against the tumor with the cooperation of helper T cells, decreasing tumor progression (21).

Currently, the use of tumor infiltrated lymphocyte quantification to stratify patients and predict survival is rising, with a score system called Immunoscore™, based on the CD3^+^ CD8^+^ T cells and memory T (CD45RO^+^) density in CRC (22–24). Alternatively, high density of FOXP3^+^ cells has been described in CRC (25), even though the relation of FOXP3^+^ Treg with CRC prognosis is still controversial (favorable prognosis (25–28); poor prognosis (29, 30). These differences can account for a heterogeneous FOXP3^+^ population, and not necessarily with a suppressor T cell phenotype (31, 32) therefore deeper studies on the function of infiltrating FOXP3+ subpopulations and their interaction with other cells of the tumor microenvironment are required to understand their role in CRC.

NK cells eliminate tumor cells lacking major histocompatibility complex class I (MHC-I) expression, by Fas/FasL pathway, by releasing perforin and granzyme, or DCs and macrophages-activating cytokines (33). MHC-I loss of expression is a common alteration in CRC (34). However, CRC tissue shows low NK cell content compared to adjacent normal tissue, independent of MHC-I expression (35), proposing poor NK infiltration to the TME as a cancer evasion mechanism in immune surveillance. Non-classical MHC class I molecule expression avoid NK antitumor function, such as human leukocyte antigen HLA-E, binding to NK cell inhibitory receptors and suppressing cytotoxic activity in CRC (36). Furthermore, peripheral NK cells from CRC patients have a dysregulated phenotype with decreased cytotoxic function, thus allowing tumor cells dissemination (37).

Tumor-associated macrophages (TAMs) are usually associated with M2-profile macrophages, possess immunosuppressive properties, promote tumor progression, and, M2-TAMs marker expression is a poor prognostic factor in CRC (38).

Other essential cells in the tumor microenvironment are myeloid-derived suppressor cells (MDSCs), that probably through T reg cell induction, in addition to NK and T effector cell suppression, inhibit antitumor responses (39). In CRC their increase in both tumor and peripheral blood correlates with stage and metastasis (39, 40), revealing their immunosuppressive activity.

Additionally, tumor associated neutrophils (TAN) appear to be an important component of tumor-infiltrating cell populations in CRC. Indeed, high TAN content is associated with improved survival in CRC patients (41–43) possibly indicating a positive response to 5-FU-based chemotherapy (44). Neutrophils in the tumor microenvironment can also promote anti-tumor responses mediated by macrophages (45) or CD8+ T cells (42), although contradictory results have been reported in mouse models. In mice with inducible colon adenoma, T‐cell suppression is mediated by neutrophil‐secreted metalloproteinase activation, thus considered an immunosuppressive mechanism in CRC (46). Moreover, neutrophils promote hepatic metastasis growth and angiogenesis mediated by fibroblast growth factor 2 (FGF2) (47). Additionally, in a model of colitis-associated colorectal cancer (CAC), an anti-neutrophil antibody reduced the number of tumors and intracolonic neutrophils infiltration along with MMP-9 mRNA expression, suggesting TANs promote tumor development (48).

Contradictions between murine models and human sample data possibly associate with differing neutrophil functions and biology, along with tumor formation, as mice form faster and do not fully mirror human tumor development stages and cellular interactions (49). Consequently, this consideration should be taken with studies from murine-human models.

There exists vast information regarding immunity in CRC, revealing a complex interaction between components of the immune system and the tumor. The immune system plays a double role, at the beginning and in the development of CRC, where immune-tumor interactions present opportunities for creating treatments preventing tumor development or improving current treatments.

## Gut Microbiome

A healthy gut microbiome varies according to the high grade of interindividual differences, influenced by diet, lifestyle, age, gender, and geography. In 2018, the International Life Sciences Institute (ILSI) of North America workshop concluded that “mechanistic links of specific changes in gut microbiome structure with function or markers of human health are not yet established” (50). However, there is consensus that the healthy gut microbiome goes through a stable and resilient equilibrium state (51) with high species diversity (50–52) predominantly composed of Bacteroidetes, Firmicutes, and Actinobacteria, exhibiting differences in distribution between mucosal-to-luminal and proximal-to-distal (53–55). The gut microbiota is influenced by the birth mode, breastfeeding (56), gender (hormones) (57, 58), pregnancy (59, 60), lifestyle (e.g. sedentary or sports practice) (61), diet (52), age (62), among others. Furthermore, diet patterns enriched in animal protein/fat or carbohydrates are associated with Bacteroides and Prevotella enterotypes, respectively (63). The above was confirmed by the fecal microbiome characterization in healthy subjects (children and adults) from Amazonas of Venezuela, rural Malawi, and the US metropolitan area revealed prominent differences between age groups and between rural and urban cohorts (52).

### Dysbiosis in Sporadic Colorectal Cancer

The first reports revealing CRC-associated dysbiosis date from 2011, which is related to the expansion of next generation sequencing (NGS) techniques (64–66) and highlighting the overrepresentation of the anaerobic bacterium *Fusobacterium nucleatum* in CRC (65, 66).

CRC patient gut microbiota differs from healthy individuals, highlighting a reduced diversity (67) at fecal and tumor levels (68), with the phyla *Proteobacteria*, *Fusobacteria*, and *Lentisphaerae* enrichment in fecal samples, and *Firmicutes* and *Actinobacteria* reduced (67). The bacterial genera *Fusobacterium, Peptostreptococcus, Porphyromonas, Prevotella, Parvimonas, Bacteroides*, and *Gemella*, are prominently enriched in CRC (69) and in contrast, the genera *Roseburia, Clostridium, Faecalibacterium* and *Bifidobacterium* decreased (68).

A metagenomics study of fecal samples from patients with CRC showed a stage-dependent microbiota variation, with some species increasing in abundance through tumor development (*Fusobacterium nucleatum, Solobacterium moorei, Peptostreptococcus stomatis, Peptostreptococcus anaerobius, Lactobacillus sanfranciscensis, Parvimonas micra* and *Gemella morbillorum)*, and some species stage-specific. The *Atopobium parvulum, Actinomyces odontolyticus, Desulfovibrio longreachensis* and *Phascolarctobacterium succinatutens* species increased only in early stage S0, in contrast, *Colinsella aerofaciens, Porphyromonas uenonis* and *Dorea longicatena* increased only in late stages III/IV (70). Similar studies with tissue samples demonstrated that early stages have an enrichment of *Fusobacterium, Leptotrichia, Gemella and Parvimonas*, and reduction of *Blautia* and *Bacteroides*. Furthermore, in CRC pre-tumor lesions an enrichment of *Pseudomonas veronii and E. coli* exists (71), suggesting these species have oncogenic potential.

A reduction of some bacterial species during gut tumorigenesis has also been reported. One example is *Faecalibaculum rodentium* and its human equivalent *Holdemanella biformis* belonging to the *Erysipelotrichaceae* family, showing a reduced fecal content in early phases of tumorigenesis from Apc^Min/+^ model and patients with large colorectal adenoma, respectively (72). These bacteria block tumor proliferation by mediating the production of SCFAs (see later in *Short-Chain Fatty Acids (SCFAs)* section), (especially butyrate) inhibiting HDAC in adenomas, by increasing H3 histone acetylation and downmodulating the calcineurin-NFATc3 pathway (72). Additionally, gut microbiota relates to tumor immune cell infiltration, where specific bacteria genera, such as *Alloprevotella, Treponema*, and *Desulfovibrio*, are enriched in tumors with high T cell marker (CD3) content and accompany prolonged CRC patient survival. Whereas, *Prevotella*, *Bacteroides*, and *Fretibacterium*, are overrepresented in CD3 low tumors by regulating chemokine expression from tumor cells (73).

All these antecedents suggest that intestinal microbiota can give both beneficial and adverse effects on gut physiology contributing to health or disease susceptibility. This may therefore lead to novel strategies enriching specific kinds of bacteria or metabolite production favoring anti-tumor immune cell recruitment or tumor proliferation blocking.

### Dysbiosis in Hereditary Colorectal Cancer

The evidence of the microbiota role in cancer development of hereditary CRC patients is scarce.

In the inherited condition FAP (caused mainly by a germline mutation of the *APC* tumor suppressor gene) (3), intestinal mucosa with precursor lesions (polyps) presents bacterial biofilms composed predominantly by *pks+ Escherichia coli* and enterotoxigenic *Bacteroides fragilis*. Additionally, co-colonization of both bacteria in a CRC murine model accelerates tumor development and increased mortality (associated with high IL-17 levels and DNA damage), suggesting *pks+ Escherichia coli* and enterotoxigenic *Bacteroides fragilis* act as protumorigenic bacteria in early colonic tumor development in genetically susceptible patients (74).

Fecal microbial patterns demonstrate that FAP patients carrying pathogenic APC mutations showing increased abundance of *Fusobacterium mortiferum* and a decreased representation of *Faecalibacterium prausnitzii* and *Bifidofacterium pseudocatenulatur*, compared to patients without an identified mutation (75). Moreover, increased seric metabolites (R)-3-Hydroxybutryric acid and 2-Hydroxyphenethylamine exists in patients carrying APC mutation, together with lower levels of 7-Ketocholesterol, DL-lactate, L-Pyroglutamic acid (75). Among other relationships, a positive correlation between *Faecalibacterium prausnitzii* and cortisol exists; however, more evidence is needed to clarify the association between metabolites and gut microbiota (75).

In characterizing Lynch syndrome patients’ microbiome, an fecal over-representation of *Faecalibacterium prausnitzii, Parabacteroides distasonis, Ruminococcus bromii, Bacteroides plebeius, Bacteroides fragilis* and *Bacteroides uniformis* was identified in both postoperative LS female patients with colorectal syndrome or LS extracolonic cancer, however distinct to controls (76). Microbiota’s role in early stages of carcinogenesis, was prospectively evaluated in fecal and mucosa samples from LS patients (carriers of pathogenic germline MMR mutation) and demonstrated: 1) colectomy and CRC history has the largest effect on microbiome profiles; 2) microbial changes are similar in Lynch adenoma and CRC; 3) fecal microbial transcriptional activity is a weak predictor of adenomas development. Although microbiome monitoring does not appear to be effective in early prediction of adenomas, the possibility exists of early microbiota changes in LS neoplasia (77).

### Tumorigenic Effects of Bacterial Species

Bacteria are involved in colorectal carcinogenesis through pathomechanisms such as: tissue invasion, local immune response modulation and metabolites or toxins secretion (**Table 1**). The most relevant CRC-associated bacteria are *Fusobacterium nucleatum, Peptostreptococcus ssp., Porphyromonas ssp., Prevotella intermedia, Parvimonas micra, Bacteroides fragilis* and *Gemella morbillorum* (69).

**Table 1:** Bacteria involved in colorectal carcinogenesis.

	Effect pro-tumor/relation with cancer	References
***Streptococus gallolyticus***	Expresses a collagen binding protein pil1 that confers a capacity to colonize tissue.	(78)
	Promote tumor progression *via* induction of proinflammatory mediator such as COX2 and IL1, as well as angiogenic cytokine IL8.	(79)
	Some *S. gallolyticus* strains are able to promote host cell proliferation and adhered to colon cancer cells while others are not. Those virulent strains can promote tumor development in AOM-induced mouse model of CRC.	(80)
***Enterotoxigenic Bacteroides fragilis (ETBF)***	Using a murine model, ETBF induces persistent subclinical colitis and hyperplasia.	(81)
	*B. fragilis* toxin (BFT) upregulates spermine oxidase, a polyamine catabolic enzyme, generating reactive oxygen species and thereby DNA damage.	(82)
	EBFT induces colitis and tumorigenesis *via* IL17 induction, activation of STAT3 and recruitment of polymorphonuclear immature myeloid cells on lamina propria.	(83, 84)
***pks+ Escherichia coli***	colibactin is able to induce DNA double strand breaks and chromosomal instability in human cells.	(85, 86)
***Fusobacterium nucleatum***	*F. nucleatum* invasion promotes a proinflammatory response in cell lines derived from colon cancer.	(87, 88)
	FadA allows bacteria attachment and invasion of E-cadherin-expressing cells, induction of human CRC and proinflammatory response associated with NF-kB2 upregulation.	(89)
	*F. nucleatum* avoiding NK-mediated tumor cell lysis *via* FAP2 interaction with the inhibitory NK-receptor TIGIT.	(90, 91)
***Peptostreptococcus anaerobius***	Promotes colorectal carcinogenesis through cholesterol synthesis induced by TLR2/TLR4 signaling activation and reactive oxygen species (ROS) generation.	(92)
	*P. anaerobius* adheres to the CRC cells and accelerates CRC development in APC^Min/+^ mice.	(93)
***Enterococcus faecalis***	Produce hydroxyl radical and extracellular superoxide causing DNA breaks promoting chromosomal instability and increased inflammation.	(94, 95)
	*E. faecalis*-infected macrophages induce aneuploidy and tetraploidy in colonic epithelial cells through of soluble mediator.	(96)

Next, the role of *pks^+^ Escherichia coli* and *Fusobacterium nucleatum* is clarified in colorectal cancer, both being examples of bacteria thoroughly studied with mechanisms associated to CRC carcinogenesis.

### *pks^+^ Escherichia coli*

*E. coli* is a commensal organism widely distributed along the gut, comprised by four phylogenetic groups (A, B1, B2 and D), with strains possibly linked to CRC as they produce inflammation and secrete toxins, such as cyclomodulins (toxins interfering with the eukaryotic cell cycle) (97). Among them, colibactin, is synthesized by 19 encoded genes within the 54-kilobase genomic island polyketide synthase (*pks*) (85), and relevantly *pks^+^ E. coli* are abundant in CRC and IBD patients (98).

**Figure 1:**
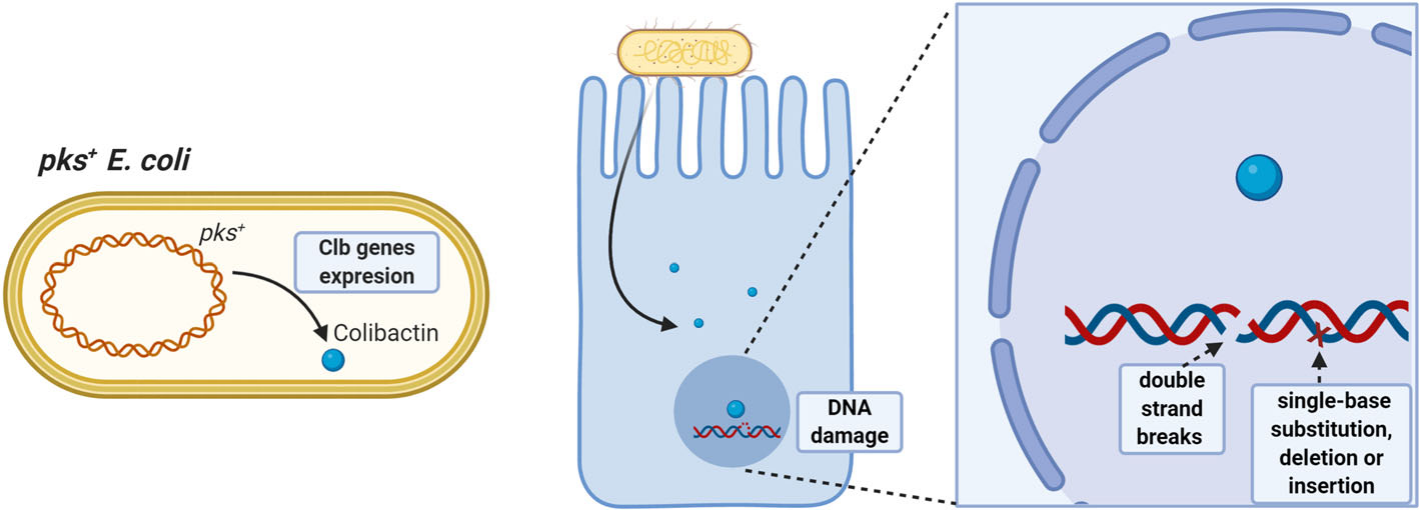
Pro-tumorigenic effects of *pks^+^ E. coli.* Strains having the pathogenicity island *pks* can synthesize colibactin toxin having oncogenic potential. Colibactin damages colonocyte DNA by inducing double-stranded DNA breaks and single-base substitution, deletion, and insertion mutations, favoring accumulation of damage and increasing the risk of malignant cell transformation.

Azoxymethane (AOM) administered to IL10^-/-^ germ-free mice colonized with *pks^+^ E. coli* or *E. faecalis* induces aggressive inflammation; however, only *pks^+^ E. coli* developed invasive adenocarcinoma, demonstrating that *E. coli*-specific factors promote colitis-associated cancer (98), as colibactin induces DNA double-strand breaks and chromosomal instability in human cells (85, 86, 99). Additionally, prolonged exposure to *pks^+^ E. coli* in human intestinal organoids induces a DNA mutational signature characterized by random single-base substitutions, deletions and insertions (100) (**Figure 1**).

Decreased tumor-infiltrating lymphocytes (TILs) in the invasive CRC margin is associated with *pks^+^ E. coli* (101). Moreover, APC^Min/+^ mice exposed to colibactin-producing *E. coli* exhibit more polyps and decreased CD3^+^ CD8^+^ T-cells than noninfected animals or infected with *pks-*lacking *E. coli* strains, suggesting colibactin induces a carcinogenic microenvironment (101).

Interestingly, microbial diversity is altered in mice receiving *pks^+^ E. coli* strain compared to non genotoxic-strain exposed mice, suggesting colibactin exert a direct effect on gut microbiota, additional to its other host effects (102).

### *Fusobacterium nucleatum*

Fusobacteria are a Gram-negative anaerobic bacilli, with *Fusobacterium nucleatum* a component of oral microbiota; although associated with oral, extraoral diseases (103), and colorectal adenomas and adenocarcinoma (65, 66, 104). Although detection rates of *F. nucleatum* in CRC patients differ widely due to methods used and samples analyzed (105) correlation between bacteria abundance and poor cancer-specific survival (106, 107) or lymph node metastasis (66) have been described, and is suggested as a prognostic marker.

**Figure 2:**
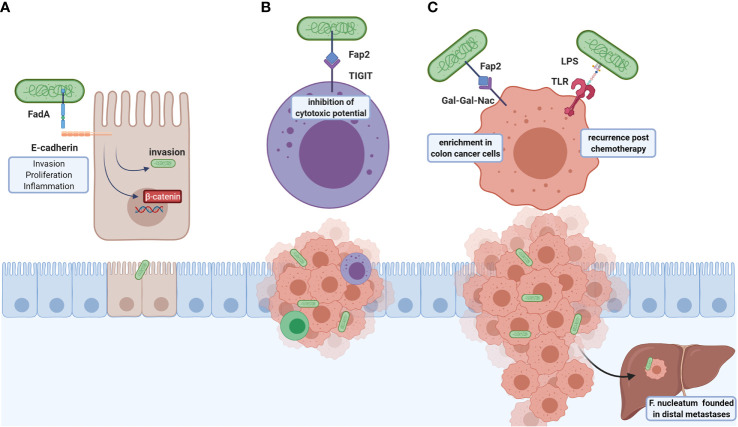
Roles of *F. nucleatum* in CRC tumoral development and metastasis. *F. nucleatum* virulence factors are FadA and Fap2: **(A)** FadA is an adhesin that binds to E-cadherin and allows bacterial invasion, which also induces the colonocyte proliferation through ß-catenin signaling and NFkB2-associated pro-inflammatory response. **(B)** Fap2 interacts with the TIGIT inhibitory receptor of NK cells resulting in cytotoxic inhibition, leading to immune evasion. **(C)** This bacterium associates with post-chemotherapy recurrence, suggested through LPS-TLR4 interaction activating autophagy, and altering chemotherapy response. Furthermore, Fap2 recognizes and binds to Gal-GalNac expressed in colorectal tumor cells; high *F. nucleatum* content found in distal metastases. The above-mentioned effects indicate that this bacterium participates in both carcinogenic and metastatic processes and may be a potential therapeutic target.

*F. nucleatum* participates in carcinogenic processes through virulent factors (**Figure 2**), highlighting the adhesion protein FadA and the autotransporter protein Fap2. FadA allows bacteria attachment and invasion of E-cadherin-expressing cells (89) and induction of human CRC cell proliferation in a FadA-dependent ß-catenin signaling activation and proinflammatory response associated with NF-kB2 upregulation (89). Another FadA function is vascular endothelial VE-cadherin removal from cell-cell junctions, increasing endothelial cell permeability, thus allowing bacteria to cross junctions (108).

Additionally, *F. nucleatum* invasion promotes a proinflammatory response mediated by p38 or MAPK signaling in HEK293T cells (87), and mediated by ROS in Caco-2 cell line (88), both pathways involved in early tumorigenesis (109). Indeed, bacterium abundance is positively correlated to local cytokine gene expression, such as TNFα and IL10, in colorectal adenomas (110). In APC^Min/+^ mouse model, *F. nucleatum* exacerbates tumorigenesis and recruits tumor-infiltrating myeloid cells (granulocytes, macrophages, DCs, and MDSCs), and shares a proinflammatory signature with *Fusobacterium*-associated human colorectal cancer, suggesting these bacteria promotes a tumor microenvironment favoring neoplasia progression (111). *F. nucleatum* content inversely correlates with CD3^+^ T cells density in tumor, showing modulation of immune response is another disease mechanism (112). Additionally, *F. nucleatum* is associated to immune evasion in cancer, avoiding NK-mediated tumor cell lysis *via* FAP2 interaction with the inhibitory NK-receptor TIGIT (90, 91).

*F. nucleatum* is associated with a high degree of microsatellite instability (MSI-high) and CpG island methylator phenotype in colorectal carcinoma, suggesting that this bacteria is involved in a molecular specific CRC subtype (113, 114). MSI-high CRCs tumors with high *F. nucleatum* load are more invasive, show low FOXP3^+^ density and elevated CD163^+^ cells (M2 macrophages), supporting the bacterial pro-tumoral role (115).

*F. nucleatum* is more abundant in CRC tissues in patients with recurrence post-chemotherapy, associated with TLR4/MyD88 signaling blocking and microRNAs (miRNA-4802 and miRNA-18a*) activating the autophagy pathway in response to chemotherapy (116). Moreover, *F. nucleatum* appears in distal metastases, demonstrating microbiota stability between primary tumors and metastasis. Likewise, mouse xenograft models retain viable *F. nucleatum* through successive passages, while metronidazole treatment reduces bacterial load, cancer cell proliferation and tumor growth (117). In summary, *F. nucleatum* induces tumorigenesis through several mechanisms including epithelial cell adhesion and tumor microenvironment modulation, becoming an attractive CRC prognosis biomarker.

## Metabolites Derived From Microbiota and Its Role in Cancer

Gut bacteria modulates the host biology through direct cell interaction, as well as microbial-derived metabolites. Therefore, we focus on the role of SCFAs and Polyamines and their functional impact on colon carcinogenesis and TME, illustrating microbiota–metabolite–cell interactions.

### Short-Chain Fatty Acids (SCFAs)

Microbiota communicates with the host through the generation of metabolites, being the most well-studied the short-chain fatty acids (SCFAs), including acetate, propionate, and butyrate, corresponding to fermentation products of complex polysaccharides, such as starches and fiber (118). The Bacteroidetes phylum mainly produces acetate and propionate, whereas the Firmicutes phylum butyrate (119).

SCFAs are produced in the colon and absorbed into the bloodstream to be delivered to target tissues, with host cells responding to SCFAs through receptors (GPR41/*FFAR3*, GPR43/*FFAR2*, GPR109A/*HCAR2*) or transporters (MCT1/*SLC16A1*, SMCT1/*SLC5A8*) (4). Butyrate is relevant in intestinal function being the primary energy source for colonocytes, corresponding to about 70% of total energy consumption (120).

Administration of SCFAs mixture restores intestinal epithelial cell turnover in antibiotic-treated SPF mice, previously having reduced amounts of acetate, propionate, and butyrate (121), demonstrating that SCFAs are essential in maintaining intestinal homeostasis. Accordingly, butyrate enhances M2-macrophage polarization (122), inhibits lipopolysaccharide (LPS)-induced proinflammatory cytokine expression in dendritic cells (123) and lamina propria (LP) mouse macrophages (124), and increases Treg lymphocytes in a murine model (125, 126).

Intestinal microbiota imbalance possibly relates to changes in SCFAs levels and loss of gut homeostasis (5). Patients with CRC and adenoma have reduced SCFAs stool levels (127), possibly related to reduced butyrate-producing bacteria such as *Bacteroides vulgaris*, *Bacteroides uniformis*, *Roseburia*, and the *Lachnospiracea* family (128–130).

**Figure 3:**
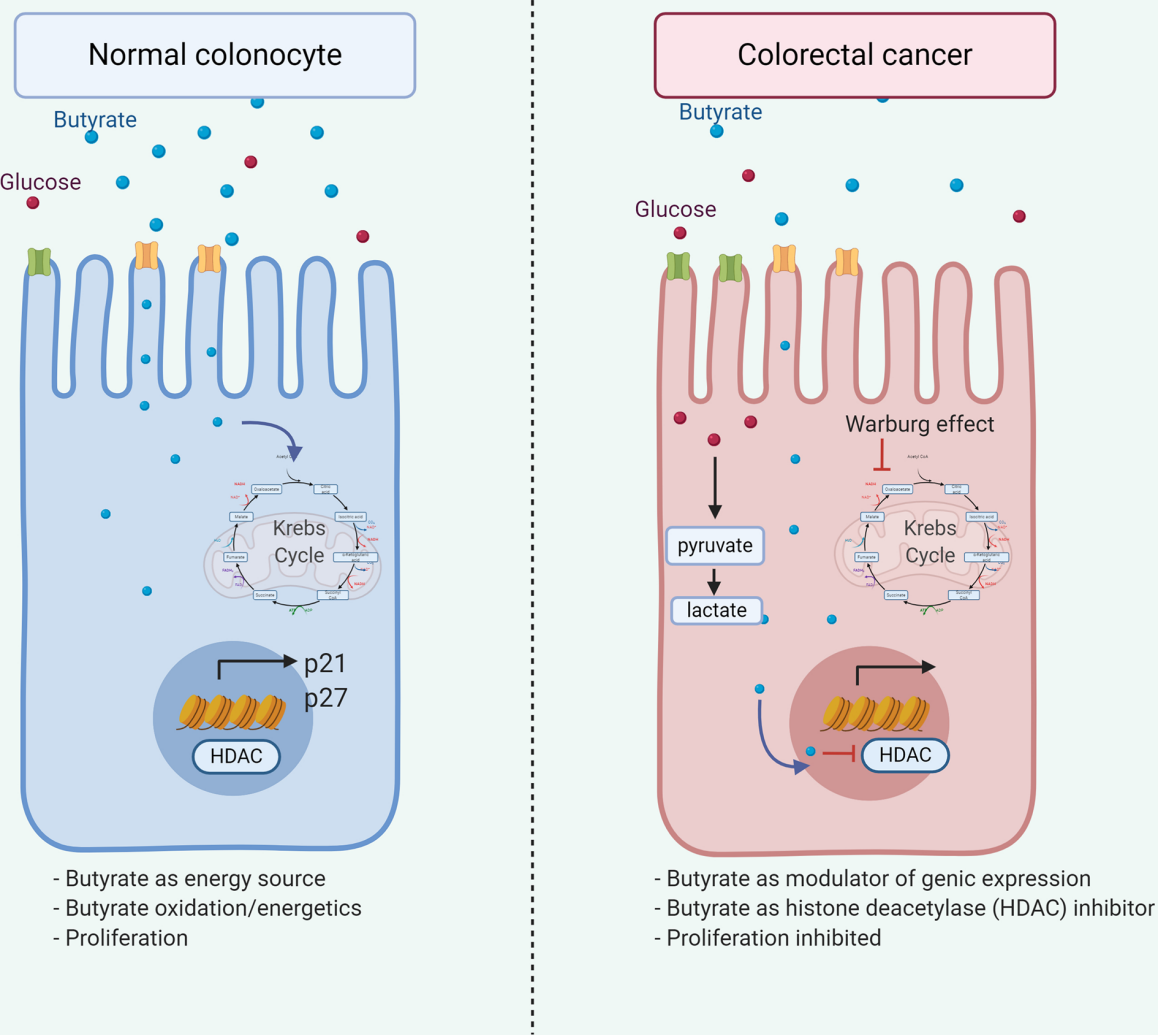
Dual role of butyrate in colorectal cancer. Butyrate exerts dual effects on normal and tumor colonocytes. In normal colonocytes, it functions as an energy source, being metabolized to acetyl-CoA in the Krebs cycle, allowing cell proliferation. Alternatively, in tumor colonocytes due to the Warburg effect, anaerobic glycolysis is the main energetic source, therefore, butyrate does not enter the Krebs cycle and available to the nucleus, modulating gene expression through the HDAC inhibition, leading to p21 and p27 downregulation and inhibiting cell proliferation, thus explaining the beneficial effect associated in cancer.

Additionally, decreased butyrate receptors GPR109A and GPR43 content in colon cancer samples (131, 132) suggests variations of microbiota or substrate impact on receptor expression on normal epithelial or transformed cells. Butyrate and propionate, but not acetate, induces crypt proliferation obtained from healthy cecal biopsies *ex vivo* (133), although, butyrate inhibits cell growth and induces colon adenoma and carcinoma cell apoptosis (134–137). These butyrate roles are associated with the Warburg effect (**Figure 3**), where butyrate generates energy by metabolizing to acetyl-CoA *via* the Krebs cycle in the normal colonocyte. However, anaerobic glycolysis is the primary energy source in cancerous colonocytes, and butyrate is available to modulate gene expression through Histone deacetylase (HDAC) inhibition and interrupting cell cycle (138) thus demonstrating how a metabolite differentially affects a cell according to its metabolic activity.

Other butyrate antitumoral effects are inhibition of epithelial-mesenchymal transition, cell proliferation, and migration in colon cancer cell lines, mediated by increased ROS levels associated with the small redox protein Thioredoxin-1 (Trx-1) downregulation (139), suggesting sodium butyrate exerts its role through Trx-1 downregulation, becoming a possible therapeutic target.

Generally, butyrate inhibits inflammation and carcinogenesis, reducing NF-kB and Wnt signaling, both active pathways in CRC (140, 141). Additionally, LPS-induced proinflammatory markers and chemokines, including CCL3 in dendritic cells is reduced after butyrate treatment (123), suggesting butyrate influences tumor microenvironment and tumor progression.

### Polyamine Metabolites

Polyamines are organic polycations involved in cell proliferation and differentiation, tissue repair, apoptosis, angiogenesis, immune response, signal transduction, and gene expression (14, 142–144). The principal polyamines are putrescine, spermidine, spermine, and cadaverine (145), participating in gut barrier function and epithelial turnover. Factors such as biosynthesis, catabolism, and transport finely control intracellular polyamines levels, maintaining a total concentration at the mM range, with free spermidine and spermine intracellular concentration corresponding respectively to 7-15% and 2-5% of the total (146).

Polyamines derive from endogenous synthesis, diet, and gut microbiota metabolism of amino-acids such as ornithine, methionine, and arginine (147), suggesting that diet influences its levels. The first step in polyamine synthesis is ornithine decarboxylation into putrescine, by Ornithine decarboxylase (ODC) (14), with oncogenes such as *Myc* (148) transcriptionally regulating ODC content and activity increased in cancer (147). Altered polyamine levels are related to cancer (146), with increased levels in CRC (149), possibly by uptake pathway and enzymatic synthesis induction (150).

Spermidine directly affects colibactin toxin synthesis by *E. coli* strains, suggesting a role of spermidine in bacterial pathogenicity and carcinogenesis (151). Polyamine levels increase throughout tumor development in the APC^Min/+^ murine CRC model, demonstrating dysregulation of its metabolic pathway could be involved in CRC development (152). Additionally, biofilms formation in colon cancer increases polyamine metabolites, suggesting polyamines produced by biofilm bacteria or the host, enhance tumor development (153). Moreover, fecal metabolomic analysis demonstrated increased amino-acids and polyamines, principally cadaverine and putrescine in CRC patients (67).

Regarding the immune response, polyamines are essential for B and T cells’ activation (154), synthesis being required to induce cytotoxic activity and T-cell proliferation *in vitro* and *in vivo* (155, 156), However, polyamines possibly have opposing roles depending on their concentrations, since increased levels in CRC interfere with anti-tumor immune function, associated to decreased adhesion molecule expression, such as CD44 and LFA-1 (157–159) and reduced cytokine production, such as IFN-γ and TNF (160–162), contributing to TME immunosuppression (163). Polyamine blocking therapy (PBT) targeting their synthesis and transport activates adaptive-dependent antitumor immune response in murine models, characterized by increased proinflammatory cytokine production, cytotoxic CD8^+^ T cell function, and decreased immunosuppressive cell levels. Consequently, increased tumor cell apoptosis leads to decreased tumor growth, indicating that blocking polyamines signaling could generate an anti-tumor immune memory and effector response, conferring protection against tumors (144, 148, 150). Moreover, polyamines inhibit macrophage polarization toward a proinflammatory M1 phenotype through ODC enzyme-dependent putrescine synthesis altering chromatin structure and preventing inflammatory gene transcription (164), thereby affecting anti-tumor responses (165).

**Figure 4:**
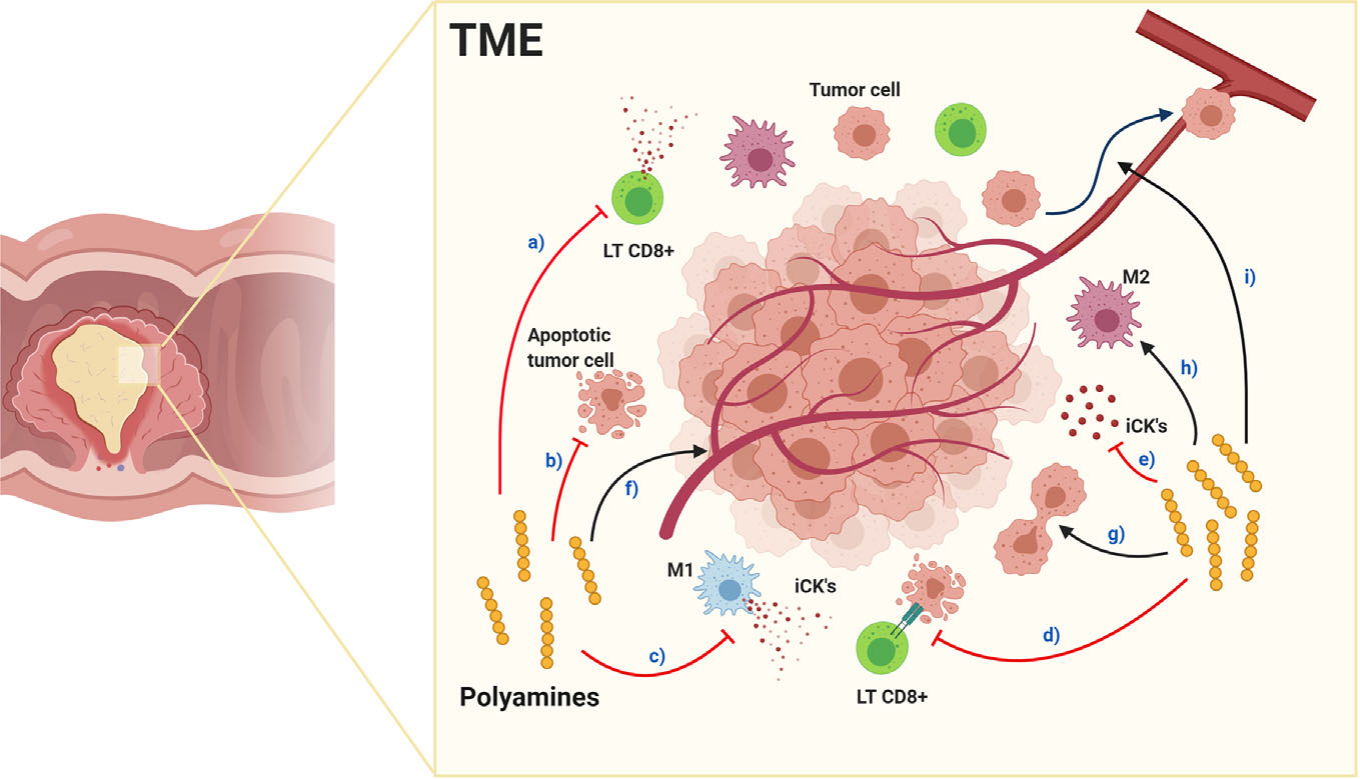
Effects of polyamines in the tumor microenvironment. Red arrows indicate inhibition and black arrows enhancement. Polyamines in TME: a) inhibit cytotoxic CD8+ LTs function and decrease quantity, inhibit: b) tumor cell apoptosis, c) macrophage polarization toward M1 pro-inflammatory phenotype, d) antitumor responses and e) pro-inflammatory cytokine production. Alternatively, polyamines enhance: f) tumor angiogenesis, g) tumor cell proliferation, h) macrophage polarization toward M2 immunosuppressive phenotype and i) tumor cell metastasis. Together, their effects produce an immunosuppressed environment facilitating tumor progression and metastasis.

Polyamines anti-inflammatory properties contribute to TME immunosuppression, (**Figure 4**), with PBT effective in inhibiting tumorigenesis, and dietary polyamine supplementation possibly benefiting aging-associated diseases (148). In TAMs, spermidine favors M1 polarization while spermine favors M2 (165), suggesting each polyamine having a unique role in normal and tumoral cells, although, exogenous spermidine treatment inhibits endogenous polyamines accumulation, tumor cell growth (166, 167) and promotes autophagy-mediated apoptosis (166). Furthermore, spermine-modified pullulan reduces immunosuppressive TME, contributing to inhibiting both tumor progression and metastasis (168). As mentioned above, endogenous polyamines could increase tumorigenesis, therefore endogenous synthesis inhibition, triggered by exogenous polyamines intake, could be a beneficial cancer treatment regulating polyamine metabolism with similar effects to PBT in the TME.

Further investigation is merited on the complex interaction between polyamine metabolism and cancer, as their targeting undoubtedly offers promising results. In conclusion, polyamines are metabolites related to microbiota and carcinogenesis, substantially contributing to TME immunosuppression, and are becoming potential therapeutic targets in CRC. Additionally, exogenous polyamine supplements could be very beneficial in combination with conventional cancer treatments.

## Influence of Diet in Microbiota and Colorectal Cancer

In 1971, Denis P. Burkitt analyzed the relation between bowel cancer frequency and fiber intake by different populations, highlighting notable differences between Western diet, where bowel cancer is more prevalent than less-developed communities. The proportion of ingested unabsorbable fiber and refined carbohydrate suggested that the diet in developed countries affected intestinal transit time, stool consistency, and bacterial microbiota-related to cancer incidence (169).

Furthermore, when comparing gut microbiota and metabolites in fecal samples from African Americans vs. native Africans, differences were found (170). Native Africans showed more abundant total bacteria, including starch fermenters and butyrate producers, along with higher SCFAs levels (171). In contrast, African Americans showed a higher abundance of microbial genes encoding for secondary bile acid production, generally associated with carcinogenic properties (172). African Americans diet is based on higher consumption of meat and fat, with lower consumption of fiber and less complex carbohydrate (170), thus suggesting the dietary influence in the production of beneficial or potentially carcinogenic metabolites.

Diet directly influences intestinal microbiota composition and metabolic activity, contributing to growing chronic diseases in the developed world, including obesity, cardiovascular disease, IBD, and cancer (173, 174). The high fat and high sugar (HF/HS) western diet have crucial implications on CRC; with a higher risk associated with red meat intake, opposed to high dietary fiber intake decreasing CRC risk (175, 176). Additionally, diet-induced intestinal inflammation (based on high plasma IL6, CRP, and TNFRSF1B levels) is associated with *F. nucleatum*-containing colorectal carcinoma in patients (177), indicating that diet alters microbiota balance.

The HF/HS diet in a mouse model induced intestinal inflammation and dysbiosis with an increase of Proteobacteria, such as *E. coli*, and decreased protective bacteria and SCFA levels (178). Furthermore, this diet gut abolishes SCFA effects on host chromatin states (histone acetylation and methylation) in colon, and extraintestinal tissues, such as liver and white adipose tissue (179). Interestingly, SCFA supplementation in germ-free mice recovers homeostatic epigenetic regulation associated with gut colonization (179).

Dietary interventions have potential effects in CRC prevention or treatment, as an adjuvant therapy. In healthy individuals, non-digestible carbohydrate (substrate for SCFA production)-enriched diets (whole grain rye flour bread/rye kernels bread + resistant starch) (180), or cooked barley kernels (181) showed beneficial effects as: a) decreased glucose and postprandial insulin levels (180, 181), b) reduce concentration of IL-6 and TNF-α (181), and c) increased satiety-inducing intestinal hormone PYY levels (180), evidencing a potential preventive effect of fiber supplementation in decreasing intestinal inflammation.

Studies of microbial interventions, using probiotics, were beneficial for CRC treatment (e.g. reducing *F. nucleatum*) (182), together with a combination of physical activity and improved dietary habits (e.g. reduction of red and processed meat or refined grain intake) in CRC survivors (183). Protocols defining the beneficial impacts of diet and physical activity in CRC patients during and after conventional therapies, and in the prevention of recurrence in CRC survivors (184) will confer benefits and should be further explored.

Considering the evidence of the influence of the diet in protecting and decreasing the risk of cancer, the 4th edition of the European Code against Cancer recommends that “people have a healthy diet to reduce their risk of cancer: eating plenty of whole grains, pulses, vegetables and fruits; limit high-calorie foods (high in sugar or fat); avoid sugary drinks and processed meat; and limit red meat and foods high in salt” (185).

## Conclusions and Perspectives

In health, microbiota participates in functions such as immunity regulation, digestion, and nutrition. As dysbiosis has been associated with diverse pathologies including CRC, microbiota could play a role in carcinogenesis, as previously demonstrated.

The immune system, microbiota and their metabolites participate in the carcinogenic process, acting differently in each tumor development stage. In sporadic CRC, specific strains have been involved in its initiation and development. Specifically, carcinogenic effects of synthesized toxins and virulence factors from *pks^+^ E. coli* and *F. nucleatum*, cause DNA damage, increased epithelial cell proliferation, and immune system modulation, thus altering the microenvironment favoring tumorigenesis. Moreover, hereditary CRC reflects dysbiosis, suggesting microbiota changes during CRC progression occur in genetically susceptible subjects.

Some diet and microbiota metabolites associated with CRC, such as SCFAs and Polyamines, directly affect tumors and TME cells, thus becoming potential therapeutic targets.

Diet modifications, such as increasing dietary fiber intake help reduce the risk of developing CRC (186). In CRC animal models, prebiotics (oligofructose-maltrodextrin) in combination with probiotics (*Lactobacillus acidophilus, Bifidobacteria bifidum*) appears beneficial, reducing tumor growth and potentially carcinogenic bacteria, while increasing butyrate concentration and NK and NKT cell number (187–190).

Natural compounds, such as curcumin, genistein (an isoflavone found in soybeans) and apigenin (present in fruits and vegetables) possibly reduce aberrant crypt foci numbers (191), increasing intestinal T and B cells (192), and reducing ODC activity and polyamine levels in CRC cell lines (193, 194).

Due to polyamines and their metabolites participating in cell proliferation, they become interesting therapeutic targets for clinical intervention of CRC. As the use of nonsteroidal anti-inflammatory Sulindac plus DMFO (an inhibitor of polyamine biosynthesis) in patients reduces adenoma recurrence (195), PBT would be a promising antitumor response alternative (144). Alternatively, nanoparticles conjugated with a polyamine analog, which increase polyamine catabolism, are a possible innovative intervention, as they induce HCT116 cell apoptosis *in vitro* and inhibit xenograft tumor growth (196).

Another option in CRC carcinogenesis is blocking-cytokine antibodies, since IL-17 and IL-23 receptor blockade inhibits colorectal tumor formation (83).

Lastly, phages are novel interventions, selectively killing potentially carcinogenic bacteria, such as *F. nucleatum* in APC^Min/+^ mice, and revert chemotherapy resistance in CRC cell lines (197).

The above therapies demonstrate a vital relationship between the microbiota, metabolites, and the immune system, highlighting the relevance of studies evaluating their interaction, and offering great potential in CRC prevention and treatment. However, more clinical trials are needed to verify their efficacy and safety.

Additional to local properties in the intestinal mucosa, the gut microbiome systemically modulate other organs (198). Likewise, gut dysbiosis has been related to obesity (199), allergy (200, 201) and extra-intestinal cancer, such as lung and pancreatic adenocarcinoma (202, 203). Enrichment of phylum *Proteobacteria* is seen as a common hallmark in diverse cancer types (204, 205), as well as a potential diagnostic dysbiosis signature and chronic metabolic disease risk, such as Type 2 diabetes mellitus and cancer (206).

Another extra-intestinal effect of gut microbiota, is seen in the gut-lung axis, where SCFAs modulate the immune system (198) and are positively associated with immunotherapy response in lung cancer patients (207). Alternatively, the gut microbiota colonizing pancreatic tumors modulate tumor growth, immune responses and thus influence patient outcome (203, 204). Therefore, the modulation of the intestinal microbiota is an interesting target to control other pathologies, not only limited to the intestine.

As can be seen from the above, delving deeper into the interactions between metabolites, the immune system, and microbiota in CRC and other pathologies, will elucidate novel therapeutic targets. Accordingly, approaches such as diet modifications, supplementation with metabolites, specific antibiotics, immune system modulation for anti-tumor response are suitable and applicable resolutions.

## Author Contributions

MH and MF wrote most of the review. DP-V and GL contributed to writing and correcting the manuscript. AW, CH, KA, MAH, and FL-K participated reviewing and critically correcting the manuscript. FL-K and MF contributed to manuscript structure and supervised the work. All authors contributed to the article and approved the submitted version.

## Funding

National Agency of Research and Development (ANID) (Fondecyt iniciación #11190990).

## Conflict of Interest

The authors declare that the research was conducted in the absence of any commercial or financial relationships that could be construed as a potential conflict of interest.
